# Gene expression profiling of cancer stem cell in human lung adenocarcinoma A549 cells

**DOI:** 10.1186/1476-4598-6-75

**Published:** 2007-11-22

**Authors:** Dong-Cheol Seo, Ji-Min Sung, Hee-Jung Cho, Hee Yi, Kun-Ho Seo, In-Soo Choi, Dong-Ku Kim, Jin-Suk Kim, Abd El-Aty AM, Ho-Chul Shin

**Affiliations:** 1 Department of Veterinary Pharmacology and Toxicology College of Veterinary Medicine, Konkuk University, 1 Hwayang-dong, Kwangjin-gu, Seoul 143-701, Republic of Korea; 2 Department of Public Health, College of Veterinary Medicine, Konkuk University, 1 Hwayang-dong, Kwangjin-gu, Seoul 143-701, Republic of Korea; 3 Department of Infectious Diseases, College of Veterinary Medicine, Konkuk University, 1 Hwayang-dong, Kwangjin-gu, Seoul 143-701, Republic of Korea; 4 Cell and Gene Therapy Research Institute, Pochon CHA University, CHA General Hospital, Seoul 135-081, Republic of Korea

## Abstract

**Background:**

The studies on cancer-stem-cells (CSCs) have attracted so much attention in recent years as possible therapeutic implications. This study was carried out to investigate the gene expression profile of CSCs in human lung adenocarcinoma A549 cells.

**Results:**

We isolated CSCs from A549 cell line of which side population (SP) phenotype revealed several stem cell properties. After staining the cell line with Hoechst 33342 dye, the SP and non-side population (non-SP) cells were sorted using flow cytometric analysis. The mRNA expression profiles were measured using an Affymetrix GeneChip^® ^oligonucleotide array. Among the sixty one differentially expressed genes, the twelve genes inclusive three poor prognostic genes; Aldo-keto reductase family 1, member C1/C2 (AKR1C1/C2), Transmembrane 4 L six family member 1 nuclear receptor (TM4SF1), and Nuclear receptor subfamily 0, group B, member 1 (NR0B1) were significantly up-regulated in SP compared to non-SP cells.

**Conclusion:**

This is the first report indicating the differences of gene expression pattern between SP and non-SP cells in A549 cells. We suggest that the up-regulations of the genes AKR1C1/C2, TM4SF1 and NR0B1 in SP of human adenocarcinoma A549 cells could be a target of poor prognosis in anti-cancer therapy.

## Background

Cancer stem cell hypothesis is the tumoral cells which have stem cell features such as self-renewal, high migration capacity, drug resistance, and aberrant differentiation which constitute the heterogeneous population of tumor [1,2]. Tissue-specific stem cells are defined by their ability to self-renew and to produce the well differentiated and functional cells within an organ. Differentiated cells are generally short-lived; in skin and blood for example, they are produced from a small pool of long-lived stem cells that last throughout the life [3-6]. Therefore, stem cells are necessary for tissue development, replacement, and repair [7]. On the other hands, the longevity of stem cells make them susceptible to accumulating genetic damage and thereby representing the growth root for cancer recurrence following treatment [8]. It was reported that some of the tumor stem cells can survive chemotherapy and support re-growth of the tumor mass [9].

Cancer stem cells (CSCs) were first identified in 1990s in hematological malignancies, mainly acute myelogenous leukemia (AML) and also in other subtypes like AML M0, M1, M2, M4, and M5, chronic myeloid leukemia (CML), acute lymphoblastic leukemia (ALL), and multiple myeloma [10,11]. CSCs are also known in solid tumors like breast, brain, lung, prostrate, testis, ovary, stomach, colon, skin, liver, and pancreas [12-17]. A character of stem cells, termed "side population (SP)", has been identified using Hoechst 33342 dye. The flow cytometric analysis makes sorting possible either to SP or non-SP cells. The SP cells have been isolated from various types of adult tissue where they demonstrate stem cell activity [18-23]. The findings of these previous studies suggest that the SP phenotype represents a common feature of stem cells.

We performed our work on human lung adenocarcinoma A549 cells (of which SP phenotype revealed several stem cell properties [24]) to identify the genes, which make the CSCs of poor prognostic phenotype and evaluate the gene expression intensities of SP and non-SP cells using oligonucleotide micro-array. The reasons why the A549 cell line was selected, because it has a relatively high proportion of SP cells compared to other cell lines [25] and is more chemo-resistant particularly to platinum drugs [26].

## Results

### The distinct gene regulations in SP cells

**Figure 1:**
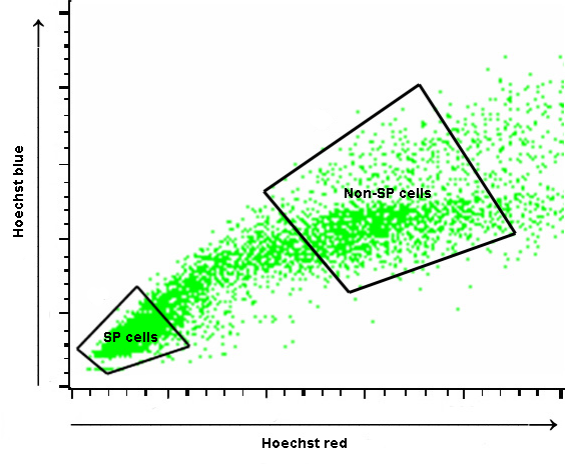
Sorting of SP and non-SP cells by FACSVantage SE.

**Figure 2:**
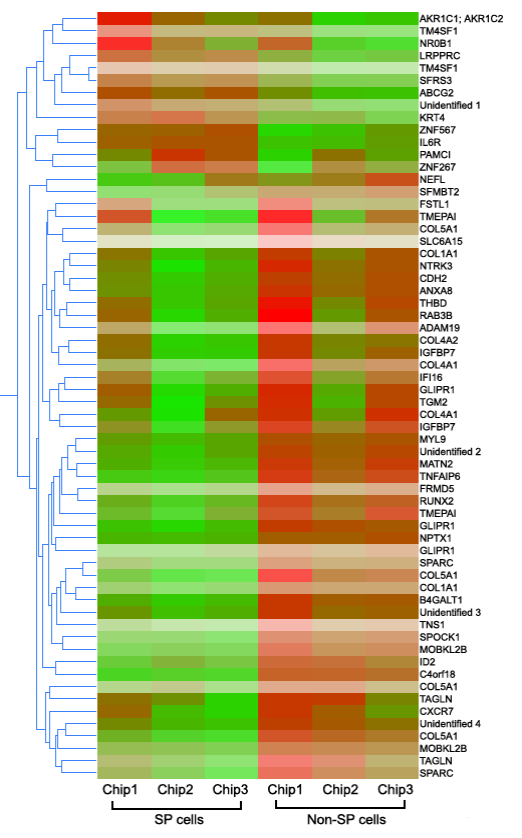
**Gene clustering of up-regulated genes in SP and non-SP cells**. After normalizing each chip to the 50^th ^percentile of the measurements taken that chip, gene-probes scored less than 0.1 either in SP or non-SP were excluded from data analysis. Only matched up-regulated genes in SP compared to non- SP cells are selected in each step of chip data analysis. The 12 genes and 46 genes were considered as up-regulated in SP and non-SP cells, respectively.

We sorted A549 cell line to SP and non-SP cells (Fig. 1) and compared the gene expression intensities of both cells. Official symbols and gene names were used in accordance with the symbol and name lists approved by HUGO (Human genome organization) Gene Nomenclature Committee (Table 1) [27]. Following data analysis, 12 genes were considered as up-regulated in SP cells (TM4SF1 has 2 probe ID) (fold changes are shown in Table 2), whereas, 49 genes were down-regulated (Fig. 2). Since we focused on distinct gene regulations, the student's *t'*-test was not employed to prevent loss of up-regulated genes in all of three chip data, though it had large chip variations.

**Table 1:** The approved gene symbols and names in reference to HUGO Gene Nomenclature

Gene symbol	Gene name
AKR1C1; AKR1C2	aldo-keto reductase family 1, member C1 (dihydrodiol dehydrogenase 1; 20-alpha (3-alpha)-hydroxysteroid dehydrogenase); aldo-keto reductase family 1, member C2 (dihydrodiol dehydrogenase 2; bile acid binding protein; 3-alpha hydroxysteroid dehydrogenase, type III)
TM4SF1	transmembrane 4 L six family member 1
NR0B1	nuclear receptor subfamily 0, group B, member 1
LRPPRC	leucine-rich PPR-motif containing
SFRS3	splicing factor, arginine/serine-rich 3
ABCG2	ATP-binding cassette, sub-family G (WHITE), member 2
Unidentified 1	adult retina protein
KRT4	keratin 4
ZNF567	zinc finger protein 567
IL6R	interleukin 6 receptor
PAMCI	peptidylglycine alpha-amidating monooxygenase COOH-terminal interactor
ZNF267	zinc finger protein 267
NEFL	neurofilament, light polypeptide 68 kDa
SFMBT2	Scm-like with four mbt domains 2
FSTL1	follistatin-like 1
TMEPAI	transmembrane, prostate androgen induced RNA
COL5A1	collagen, type V, alpha 1
SLC6A15	solute carrier family 6, member 15
COL1A1	collagen, type I, alpha 1
NTRK3	neurotrophic tyrosine kinase, receptor, type 3
CDH2	cadherin 2, type 1, N-cadherin (neuronal)
ANXA8	annexin A8
THBD	thrombomodulin
RAB3B	RAB3B, member RAS oncogene family
ADAM19	ADAM metallopeptidase domain 19 (meltrin beta)
COL4A2	collagen, type IV, alpha 2
IGFBP7	insulin-like growth factor binding protein 7
COL4A1	collagen, type IV, alpha 1
IFI16	interferon, gamma-inducible protein 16
GLIPR1	GLI pathogenesis-related 1 (glioma)
TGM2	transglutaminase 2 (C polypeptide, protein-glutamine-gamma-glutamyltransferase)
COL4A1	collagen, type IV, alpha 1
IGFBP7	insulin-like growth factor binding protein 7
MYL9	myosin, light chain 9, regulatory
Unidentified 2	CDNA FLJ44429 fis, clone UTERU2015653
MATN2	matrilin 2
TNFAIP6	tumor necrosis factor, alpha-induced protein 6
FRMD5	FERM domain containing 5
RUNX2	runt-related transcription factor 2
TMEPAI	transmembrane, prostate androgen induced RNA
GLIPR1	GLI pathogenesis-related 1 (glioma)
NPTX1	neuronal pentraxin I
GLIPR1	GLI pathogenesis-related 1 (glioma)
SPARC	secreted protein, acidic, cysteine-rich (osteonectin)
COL5A1	collagen, type V, alpha 1
COL1A1	collagen, type I, alpha 1
B4GALT1	UDP-Gal:betaGlcNAc beta 1,4- galactosyltransferase, polypeptide 1
Unidentified 3	-
TNS1	tensin 1
SPOCK1	sparc/osteonectin, cwcv and kazal-like domains proteoglycan (testican) 1
MOBKL2B	MOB1, Mps One Binder kinase activator-like 2B (yeast)
ID2	inhibitor of DNA binding 2, dominant negative helix-loop-helix protein
C4orf18	chromosome 4 open reading frame 18
COL5A1	collagen, type V, alpha 1
TAGLN	transgelin
CXCR7	chemokine (C-X-C motif) receptor 7
Unidentified 4	Transcribed locus, moderately similar to XP_517655.1 PREDICTED: similar to KIAA0825 protein [Pan troglodytes]
COL5A1	collagen, type V, alpha 1
MOBKL2B	MOB1, Mps One Binder kinase activator-like 2B (yeast)
TAGLN	transgelin
SPARC	secreted protein, acidic, cysteine-rich (osteonectin)

**Table 2:** Gene list up-regulated in SP cells compared to non-SP cells

Probe ID	Genbank	Gene symbol	Fold change (Mean ± SD)
217626_at	BF508244	AKR1C1; AKR1C2	3.11 ± 0.92
238168_at	AI760128	TM4SF1	2.77 ± 0.66
206645_s_at	NM_000475	NR0B1	2.78 ± 0.81
1557360_at	CA430402	LRPPRC	2.72 ± 0.37
215033_at	AI189753	TM4SF1	2.72 ± 0.46
232392_at	BE927772	SFRS3	2.51 ± 0.08
209735_at	AF098951	ABCG2	2.51 ± 0.37
238476_at	AA481560	Unidentified 1*	2.43 ± 0.18
213240_s_at	X07695	KRT4	2.43 ± 0.28
235648_at	AA742659	ZNF567	2.47 ± 0.75
205945_at	NM_000565	IL6R	.36 ± 0.24
210335_at	AF056209	PAMCI	2.31 ± 0.29
219540_at	AU150728	ZNF267	2.20 ± 0.27

### Validation of gene regulations

**Figure 3:**
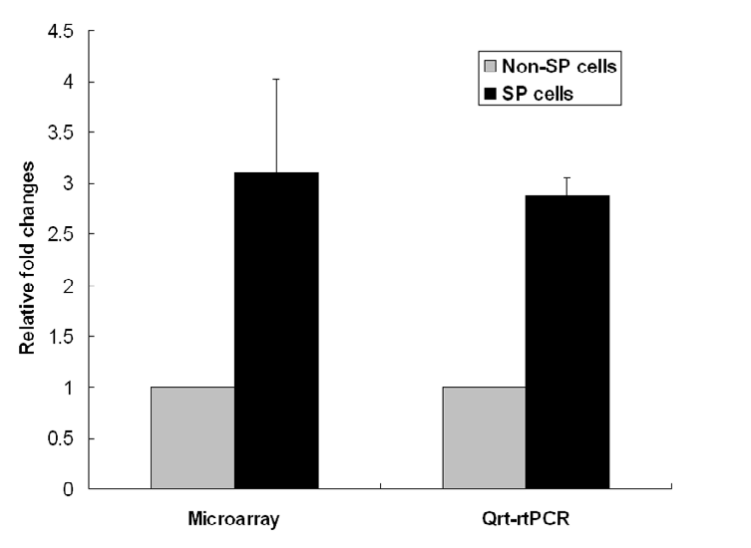
**Relative fold changes of AKR1C1/C2 gene in SP and non-SP cells**. The fold changes of AKR1C1/C2 between SP and non-SP cells were compared using GeneChip data and quantitative real time-reverse transcriptase PCR. The data (n = 3) were presented as mean ± SD.

To confirm the fold changes of *AKR1C1 *in chip data, quantitative real time – reverse transcriptase PCR was employed. The relative fold changes in SP compared to non-SP cells were 3.11 ± 0.92 and 2.88 ± 0.17 in microarray and qrt- rtPCR, respectively (Fig. 3).

## Discussion

Based on the cancer stem cell hypothesis, we assumed that the up-regulation of certain genes that are related to poor prognosis (high migration capacity or drug resistance) in SP of cancer cells could be a target for therapeutic index. In the present study, we found some genes that are related to drug resistance, such as AKR1C1/C2 and NR0B1, or cancer metastasis, such as TM4SF1, were up-regulated in SP cells of human lung adenocarcinoma A549 cell line. Furthermore, the up-regulated gene, ABCG2, has been noticed to be as an indicator for sorting SP cells by Hoechst 33342 staining [24]. It was reported that ABCG2 pumping out the drugs was associated with multi-drug resistance in many cancers [28,29] and/or effects higher levels of DNA repair and hence lowered the ability to apoptosis [30].

**Figure 4:**
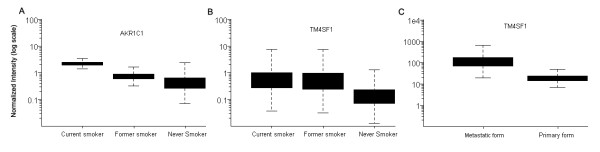
**The up-regulation of AKR1C1 and TM4SF1 in smokers (derived from GEO public database)**. AKR1C1 (A) and TM4SF1 (B) were increased in current smoker compared to former or never smokers. TM4SF1 was increased in metastatic form compared to primary form of colon cancer (C).

AKR1C belongs to a superfamily of monomeric, cytosolic NADP(H)-dependent oxidoreductases that catalyzes the metabolic reduction and either activate or inactivate several xenobiotics [31,32]. In humans, at least four isoforms of AKR1C (AKR1C1~4) have been identified [33]. AKR1C1/AKR1C3 was known to inactivate progesterone, which could alter the progesterone/estrogen ratio in certain cancers [34,35]. Additionally, AKR1C1/C2 inhibitors were reported as potential anti-neoplastic agents [36,37]. The high expression of AKR1C was considered as a poor prognostic factor in patients with non-small-cell lung cancer [38], and it was enriched in hepatocellular carcinoma than normal hepatic cells [39]. A previous study evaluated the relationship between the AKR1C and drug resistance and revealed that the over expression of AKR1C1/C2 led to drug resistance in non-small-cell lung cancer cells [26]. A public database (Gene Expression Omnibus (GEO), NCBI) [40] has shown significant up-regulation of AKR1C1 in smokers (Figure 4A). Similar trends were also reported in lung cancer patients [41,42]. This means that smoking can alter the regulation of certain genes related to poor prognosis such as AKR1C1. Moreover, AKR1C1 was suggested as a marker of stem-like cells in thyroid cancer cell lines, though it was not proved by the authors [41].

From the data of GEO, the TM4SF1 gene, which is believed to be involved in cancer invasion and metastasis [44] was also up-regulated in smokers (Figure 4B), and metastatic form of colon cancer patients compared to primary form of colon cancer patients (Figure 4C). TM4SF1 was also suggested as a possible marker of stem-like cells in thyroid cancer cells [41]. However, we could not find out the GEO data that match to tumor genesis of the up-regulated NR0B1, though up-regulated NR0B1 gene was required for the transformed phenotype of certain sarcoma [45].

## Conclusion

It is still unclear how the cancer stem cells express poor prognostic phenotype in cancer, but we found for the first time that several genes related to chemo-resistance and metastasis are up-regulated in SP cells compared to non-SP cells. Therefore, enrichment of AKR1C1/C2, TM4SF1 and NR0B1 in SP of A549 cells might be a target of poor prognosis in cancer therapy.

## Methods

### Cell culture

A549 cells, a representative human lung adenocarcinoma cell line, were obtained from American Type Culture Collection (ATCC, VA, USA). The cells were cultured on F-12K nutrient mixture, Kaighn's modification (1×) liquid (Gibco, CA, USA) supplemented with 10% fetal bovine serum (FBS) (Genetron Life Technology, INC., CA, USA) and 1% of penicillin/streptomycin (P/S) (Gibco, CA, USA) on standard plastic tissue culture dishes (SPL Life Sciences, Seoul, Republic of Korea) and incubated in an atmosphere of 95% air/5% CO_2 _at 37°C.

### Fluorescence activated cell sorting (FACS) for SP and non-SP cells

The cells were detached with trypsin (Gibco, CA, USA), washed with phosphate buffer solution (PBS)/2% FBS, and resuspended at 1 × 10^6 ^cells per ml in pre-warmed Hanks' balanced salt solution (HBSS; Gibco, CA, USA)/10% FBS. Hoechst 33342 dye (Sigma, St. Louis, MO, USA) was then added to this portion (final concentration: 5 μg/ml), and incubation continued for 90 min at 37°C. After washing with PBS/2% FBS, the cells re-suspended in ice-cold HBSS/10% FBS were labeled with 2 μg/ml propidium iodide (Sigma, St. Louis, MO, USA) to distinguish live from dead cells prior to analysis. SP analysis and sorting were done using a FACSVantage SE (BD Biosciences, CA, USA). The Hoechst 33342 dye excited at 350 nm using UV laser and the DM610 SP was used to distinguish the red from the blue fluorescence signals. The EF675-LP (Hoechst Red) and BP450/20 nm (Hoechst Blue) filters were then installed in front of the PMT detector.

### RNA isolation and oligonucleotide microarray analysis

We prepared total RNA from approximately 2 × 10^6 ^SP or non-SP cells with Qiagen RNeasy Mini kit (Qiagen, CA, USA) according to the instruction of the manufacturer. The RNAs were subjected to GeneChip^® ^expression array in full commercial service with two-cycle target labeling (SeouLin Bioscience, Seoul, Republic of Korea). Briefly, cDNA were synthesized from total RNA using T7-Oligo (dT) primers. Using that cDNA, biotinylated cRNA was then synthesized. Fifteen μg of the labeled cRNA was hybridized to a Human Genome U133 Plus 2.0 Array (Affymetrix, CA, USA). Array image was scanned and analyzed using Genechip operating software (GCOS) (Affymetrix, CA, USA).

### Quantitative real-time rt-PCR (qrt-rtPCR)

To validate the fold changes in the expression intensity of SP and non-SP cells in microarray data, we performed qrt-rtPCR using SYBR Premix Ex Taq Perfect Real Time kit (TaKaRa Bio, Ohtsu, Japan) in a SmartCycler (Cepheid, CA, USA) according to the manufacturer's instruction. The cycle threshold value, which was determined using second derivative, was used to calculate the normalized expression of the indicated genes using Smartcycler Software (Cepheid, CA, USA). The following primer pairs were used: *GAPDH *(as an internal control); F-primer 5'CGACCACTTTGTCAAGCTCA3' and R-primer 5'AGGGGAGATTCAGTGTGGTG3', *AKR1C1*;*AKR1C2*; F-primer 5'GTGGAAGCTGACCAGGTTGT3' and R-primer 5'AAGCCGTGTTCTTTCTGCTG3'.

### Microarray data analysis

We used GeneSpring GX 7.3.1 software (Agilent Technologies, CA, USA) to normalize and analyze the microarray data. Following normalization of each chip to the 50^th ^percentile of the measurements taken that chip, probes (genes) scored less than 0.1 both in SP and non-SP cells were excluded from the data analysis. Only matched up-regulated genes in SP and non-SP cells were selected from each step of chip (gene expression microarray) data analysis. More than 2 fold changes in all chip data were considered as up-regulated. Standard curve method was used to analyze qrt-rtPCR for confirmation of fold changes in chip data.

## Competing interests

The author(s) declare that they have no competing interests.

## Authors' contributions

DS and HS conceived the study. DS, JS, HC and HY carried out the sample preparation, gene expression analyses and quantitative real time RT-PCR. DK contributed to stem cell preparation. KS, IC, JK, AMAE and HS participated in the design, reviewed all data, and contributed in the preparation of the manuscript. All authors read and approved the final manuscript.
